# Metformin improved health-related quality of life in ethnic Chinese women with polycystic ovary syndrome

**DOI:** 10.1186/s12955-016-0520-9

**Published:** 2016-08-24

**Authors:** Huang-TzOu, Pei-Chi Chen, Meng-Hsing Wu, Chung-Ying Lin

**Affiliations:** 1 Institute of Clinical Pharmacy and Pharmaceutical Sciences, College of Medicine, National Cheng Kung University, 1 University Road, Tainan, 7010 Taiwan; 2 Department of Obstetrics and Gynecology, College of Medicine, National Cheng Kung University and Hospital, Tainan, Taiwan; 3 Department of Rehabilitation Sciences, Faculty of Health and Social Sciences, The Hong Kong Polytechnic University, Hung Hom, Hong Kong

**Keywords:** Metformin, Polycystic ovary syndrome, Chinese, Health-related quality of life, Overweight, Hyperandrogenism

## Abstract

**Background:**

Few studies have assessed whether the amelioration of the clinical signs of polycystic ovary syndrome (PCOS) achieved by treatment leads to improvement in the health-related quality of life (HRQoL) of patients. This study was aimed to examine the HRQoL of ethnic Chinese women with PCOS who received metformin treatment.

**Methods:**

This prospective study was conducted at a medical center in Taiwan. Study participants aged 18–45 years were diagnosed as having PCOS according to the Rotterdam criteria, and all received metformin treatment. Their HRQoL was assessed using generic (WHOQOL-Bref) and PCOS-specific (Chi-PCOSQ) instruments. Mixed effect models were used to examine the effects of metformin on repeatedly measured HRQoL. Additional analyses using stratified patients characteristics (overweight vs. normal; hyperandrogenism vs. non-hyperandrogenism) were done.

**Results:**

We recruited 109 participants (56 % were overweight, 80 % had hyperandrogenism). Among the domain scores of WHOQOL-Bref, the psychological domain score was the lowest one (12.64 ± 2.2, range 4–20). Weight (3.25 ± 1.59, range 1–7) and infertility (3.38 ± 1.93, range 1–7) domain scores were relatively low among the domain scores of Chi-PCOSQ. Overweight and hyperandrogenic patients had significantly lower HRQoL as compared with those of normal weight and non-hyperandrogenic patients, respectively. Metformin significantly improved the physical domain of WHOQOL-Bref (*p* = 0.01), and the infertility (*p* = 0.043) and acne and hair loss aspects (*p* = 0.008) of PCOS-specific HRQoL. In the subgroup analysis, significantly improved HRQoL following metformin treatment appeared for only overweight and hyperandrogenism subgroups.

**Conclusions:**

Metformin might improve health-related quality of life of polycystic ovary syndrome women by ameliorating psychological disturbances due to acne, hair loss and infertility problems, especially for overweight and hyperandrogenic patients.

## Background

Health-related quality of life (HRQoL) is generally defined as the functional effect of a clinical condition and/or its treatments upon a patient, which is subjective and multidimensional, including physical function, psychological state, and social interactions [1]. With medical advances that have improved life expectancy, population health is measured not only on the basis of saving lives but also in terms of improving the quality of life. The ultimate goal of healthcare is to improve, restore, or preserve the quality of life of patients [2]; survival per se may no longer be perceived to be only important outcome. Hence, in addition to traditional measures of health (e.g., survival), HRQoL is an important indicator that captures the burden of illness. For chronic illnesses or clinical conditions for which there is no cure, it is critical to provide therapy that makes patients feel better. To assess HRQoL, the degree to which the disease or its treatment influences the patient’s life is quantified from an individual’s perspective. Assessing HRQoL helps healthcare providers understand whether patients are satisfied with their health and associated treatments. Also, HRQoL is important to consider when evaluating various symptom management plans [3] and disease treatments [4], especially when they provide similar effects on life expectancy. According to U.S. Food and Drug Administration’s guidance for industry, HRQoL can be used as a clinical outcome to claim the effect of treatment [5].

Polycystic ovary syndrome (PCOS) is the most common endocrine disorder in reproductive-aged women [6]. Clinical presentations associated with PCOS, such as overweight/obesity, hirsutism, acne, hair loss/ androgenic alopecia, oligomenorrhea, amenorrhea and infertility and can lead to mood disturbances, affect the emotional well-being as well as sexual satisfaction of women, and cause a reduction in the HRQoL of patients [7, 8]. Obesity, clinical signs of hyperandrogenism (i.e., acne, hair loss), and infertility are the main contributors to psychological morbidity [9–11]. The HRQoL of women with PCOS has been investigated in several studies for some countries [7, 12, 13]; however, data on the HRQoL of Chinese women with PCOS is limited. It has been recognized that clinical representations of PCOS vary with culture and ethnicity [14], and may thus have different impacts on HRQoL. For example, the prevalence of hirsutism and obesity in Chinese women with PCOS appears to be lower than that from Caucasians patients [14]; contrarily, acne and hair loss were common problems reported in ethnic Chinese women with PCOS [15]. Therefore, assessing the impact of PCOS on the HRQoL of patients across ethnic groups is important.

Metformin, which increases insulin sensitivity, is one of common treatments for PCOS in Taiwan. Some studies showed that metformin improves the body weight, insulin sensitivity, acne, hirsutism, and menstrual cycle of women with PCOS, and that the effects of metformin may vary depending on a patient’s characteristics (i.e., obesity, hyperandrogenism) [16–19]. However, other research found that, among obese women with PCOS, metformin may not lower body weight or improve the menstrual cycle and weight loss alone through lifestyle modifications improves menstrual function [20]. Of notice, previous research primarily focused on clinical effectiveness of metformin [16–20], but only a few studies [7, 21] have determined whether the amelioration of the clinical signs of PCOS achieved by treatment leads to improvement in the HRQoL of patients. Therefore, this study aimed to assess the impact of PCOS on the HRQoL of ethnic Chinese women with PCOS and the effects of metformin on the HRQoL of PCOS patients.

## Methods

This was a prospective observational study. Before commencement of the study, permission was obtained from the Institutional Review Board of National Cheng Kung University Hospital, Tainan, Taiwan (A-ER-103-287).

### Participants

All participants were recruited from the Department of Obstetrics and Gynecology at National Cheng Kung University Hospital during February to August, 2015. They met the following inclusion criteria: (1) aged 18–45 years, (2) diagnosed with PCOS according to the Rotterdam criteria, defined as the presence of at least two of the following three criteria: (i) oligo-anovulation (a cycle length of > 35 days or amenorrhoea), (ii) clinical hyperandrogenism (hirsutism recorded as m-FG score of ≥ 6 with/without acne or androgenic alopecia) and/or biochemical hyperandrogenism (total testosterone level of more than 0.95 ng/mL), and (iii) polycystic ovaries (≥12 follicles measuring 2–9 mm in diameter, or ovarian volume > 10 ml in at least one ovary) [22], and (3) competent in Mandarin Chinese. We excluded those: (1) diagnosed with similar clinical presentations (e.g., hyperprolactinemia, thyroid dysfunction, Cushing syndrome), (2) diagnosed with diabetes or had fasting plasma glucose ≥ 126 mg/dL or 2-h glucose ≥ 200 mg/dL before PCOS diagnosis, (3) taking any medications that may influence insulin level or contraceptive pills at 3 months before PCOS diagnosis, (4) suffered a major traumatic event at least 6 months prior to the data collection (i.e., divorce, separation, or death of intimate partner or relatives). This is because the people experiencing these life events are likely to have negative emotions (e.g., depression, sad, anxiety), which may affect their psychological well-being [23–25]. All participants gave a written informed consent regarding their willingness to participate in the research. The second author used the questionnaires to face-to-face interview each participant at every time when they returned for a visit during 6 months of metformin treatment. Patients were scheduled for a following visit within 1 to 2 months. The author confirmed that all participants had completed all study questionnaires and demographic questions measuring age, gender, residence, highest education, disease duration, comorbidities, and exercise behavior. Routine physical examination (i.e., weight, height, PCOS-specific physical appearance: acne, hirsutism) was conducted at each visit. Overweight was defined using body mass index (BMI) calculated by weight and height, and a BMI ≥25 kg/m^2^ suggests overweight. A blood test was taken at each visit to determine hormone and glycemic levels.

Once PCOS was diagnosed, all patients were treated with metformin (500 mg TID). Patients who can not tolerate the immediate-release formulation (i.e., due to gastrointestinal intolerance side effects) could reduce the dose of metformin (from TID to BID) or were switched to extended-release metformin.

### Study measurements

The following questionnaires were administered to each participant before, during, and after 6 months of metformin treatment.

### Health-related quality-of-life questionnaire for women with polycystic

Ovary Syndrome (PCOSQ) [26] is a disease-specific HRQoL questionnaire that contains 26 questions using a seven-point rating scale (1: maximum impairment and 7: no impairment of HRQoL) in the following five domains: emotions (7 items), hair growth (5 items), body weight (5 items), infertility (5 items), and menstruation (4 items). The psychometric properties of the PCOSQ showed good test-retest reliability (all intraclass correlation coefficients > 0.8), acceptable internal reliability (all Conbroach’s α values > 0.7), and satisfactory concurrent validity with the SF-36 [27]. The present study used a Chinese version of PCOSQ (Chi-PCOSQ), which was recently developed by Ou et al. [15]. Its score was shown to be reliable and valid in a sample of Chinese-speaking women with PCOS.

WHOQO-Bref [28] is a short version of the World Health Organization Quality of Life (WHOQOL)-100. It has 26 items. The Taiwan version of WHOQOL-Bref additionally includes two domestic items. There are 26 items distributed into four domains: physical health (7 items), psychological health (6 items), social relations (4 items), and environment (11 items). Items are rated on a 5-point Likert scale (low score of 1 to high score of 5). The mean score for each domain is calculated, resulting in a mean score per domain that is between 4 and 20. The total score of WHOQOL-Bref is the sum of all domain scores; it ranges from 16 to 80, with a higher score indicating better quality of life. Internal consistency (Cronbach’s α = 0.70–0.91), test-retest reliability (*r* = 0.76–0.80), and construct validity (comparative fit index = 0.89) have been established for the Taiwan version scores [29].

MMSA-8 In order to account for the effect of medication behavior, the Morisky 8-item medication adherence scale (MMAS-8) [30] was applied to measure medication adherence. The MMAS-8 is one of the most commonly used self-report adherence questionnaires. The Chinese version of MMAS-8 has been validated among a convenience sample of 176 patients in China [31]. The scale showed acceptable internal consistency (Cronbach’s α = 0.77) and test-retest reliability (*r* = 0.88), and good construct validity.

### Statistical analyses

Descriptive analyses were used to present the demographics of the study sample and the WHOQOL-Bref and Chi-PCOSQ total and domain scores. Repeated measures analysis of variance (ANOVA) was performed to detect the changes in the HRQoL outcomes along with treatment time between subgroups. Mixed effect models were applied to assess the effects of metformin on repeated outcomes measures (including total and domain scores of WHOQOL-Bref and Chi-PCOSQ). Study patients were stratified by BMI (overweight vs. normal) and clinical and/or biochemical hyperandrogenism. Mixed effect models were applied within each subgroup to detect significant change in the HRQoL following metformin treatment. The SAS 9.4 was utilized for all aforementioned analyses.

## Results

A total of 109 eligible women were enrolled in the study, with average follow-up time of 5.18 (±1.06) months. There were 83 patients who completed 6 months of follow-up, 12 patients who were lost follow-up, 11 patients who got pregnancy, and 3 patients who switched to oral contraceptive pills. The mean age of the participants was 28.3 years; 56 % of them were overweight and 80 % had hyperandrogenism. The detailed baseline characteristics are presented in Table 1.

**Table 1:** Demographics of study population

Characteristics	All (*n =* 109)	Subgroups			
		Normal weight (BMI < 25 kg/m^2^) (*n =* 48)	Overweight (BMI ≧ 25 kg/m^2^) (*n =* 61)	Non-hyperandrogenism (*n =* 22)	Hyperandrogenism (*n =* 87)
	Mean ± SD	Mean ± SD	Mean ± SD	Mean ± SD	Mean ± SD
Age (years)	28.3 ± 5.1	27.12 ± 4.47	29.3 ± 5.47	27.6 ± 4.9	28.0 ± 5.2
BMI (kg/m^2^)	26.8 ± 6.4	21.1 ± 1.9	31.6 ± 4.6**	23.8 ± 4.9	27.6 ± 6.5*
Systolic BP (mmHg)	121.9 ± 16.3	113.1 ± 12.3	129.2 ± 15.7**	110.3 ± 11.3	125.2 ± 16.1*
Diastolic BP (mmHg)	76.4 ± 13.7	69.8 ± 9.7	81.9 ± 14.3**	69.4 ± 9.9	78.4 ± 14.1*
mF-G score	3.2 ± 2.1	2.8 ± 2.3	3.5 ± 1.9	2.5 ± 1.5	3.4 ± 2.2
Hormones	Mean ± SD	Mean ± SD	Mean ± SD	Mean ± SD	Mean ± SD
TSH (μIU/mL)	1.72 ± 0.91	1.40 ± 0.72	2.00 ± 0.87*	1.49 ± 0.74	1.79 ± 0.95
LH (mIU/mL)	8.78 ± 4.82	11.02 ± 5.40	6.78 ± 3.17**	11.28 ± 5.80	8.01 ± 4.25*
FSH (mIU/mL)	5.29 ± 2.07	5.50 ± 1.93	5.10 ± 2.20	5.24 ± 2.12	5.31 ± 2.08
E2 (pg/mL)**	40.13 ± 41.74	36.41 ± 27.37	43.56 ± 51.73	40.21 ± 31.34	40.11 ± 44.56
PRL (ng/mL)	9.12 ± 3.92	8.90 ± 3.59	9.31 ± 4.24	9.48 ± 4.01	9.01 ± 3.93
TT (ng/mL)	0.58 ± 0.32	0.57 ± 0.37	0.59 ± 0.27	0.54 ± 0.24	0.59 ± 0.34
Glycemic parameters	Mean ± SD	Mean ± SD	Mean ± SD	Mean ± SD	Mean ± SD
2-h glucose (mg/dL)	113.2 ± 29.0	103.6 ± 26.9	121.2 ± 28.6*	105.4 ± 24.0	115.4 ± 30.1
2-h insulin (μIU/mL)	89.8 ± 91.1	46.2 ± 25.3	126.9 ± 109.2**	52.8 ± 50.5	100.0 ± 97.3*
Clinical presentation	n (%)	n (%)	n (%)	n (%)	n (%)
Hirsutism	12 (16.2)	5 (14.7)	7 (17.5)	0 (0.0)	12 (20.7)
Acne	36 (48.6)	16 (47.1)	20 (50.0)	0 (0.0)	36 (62.1)**
Weight gain	33 (44.6)	11 (32.4)	22 (55.0)	5 (31.3)	28 (48.3)
Hair loss	40 (54.1)	15 (44.1)	25 (62.5)	0 (0.0)	40 (69.0)**
Impaired glucose intolerance	14 (18.9)	4 (11.8)	10 (25.0)	1 (6.3)	13 (22.4)
Insulin resistance	54 (73.0)	20 (58.8)	34 (85.0)*	8 (50.0)	46 (79.3)*

*Abbreviations*: *BMI* body mass index, *BP* blood pressure, *LH* luteinizing hormone, *FSH* follicle stimulating hormone, *E2* estradiol, *PRL* prolactin, *TT* total testosterone, *mF-G* modified Ferriman-Gallwey, *SD* standard deviation

*p < 0.05, **p < 0.001

The changes of HRQoL were revealed by the total and domain scores of both WHOQOL-Bref and Chi-PCOSQ within the period of metformin treatment (Table 2). Overall, PCOS patients had the lowest score in the psychological aspect of HRQoL, with similar trends found within subgroups. These imply higher impact of psychological disturbances to patients’ quality of life. Overweight patients had significantly lower HRQoL (relatively poorer quality of life) as compared to that of normal weight patients in WHOQOL-Bref scores. Repeated measures ANOVA indicated that the changes in psychological and social domain scores along with treatment duration between overweight and normal weight subgroups were significantly different (*p* = 0.027 and *p* = 0.016 for psychological and social domains, respectively). Hyperandrogenic patients had significantly lower HRQoL as compared to that of those without hyperandrogenism. The changes in total score and physical, psychological, and social domain scores along with treatment time between hyperandrogenism and non- hyperandrogenism subgroups were significantly different (repeated measures ANOVA showed *p* = 0.014, 0.012, 0.013, and 0.015 for total score and physical, psychological, and social domain scores, respectively).

**Table 2:** Change in HRQoL in Chinese women with PCOS along with metformin treatment duration

WHOQOL-Bref	Total Mean (SD)	Subgroups			
		Normal weight Mean (SD)	Overweight Mean (SD)	Non- hyperandrogenism Mean (SD)	Hyperandrogenism Mean (SD)
Total scores (16–80)					
Visit 1 (baseline)	55.05 ± 6.75	56.51 ± 6.27	53.80 ± 6.97	58.34 ± 6.27	54.14 ± 6.65
Visit 2 (1st -2nd month of treatment)	56.19 ± 7.38	57.54 ± 7.15	55.05 ± 7.48	60.87 ± 6.98	55.90 ± 7.02
Visit 3(3rd- 4th month of treatment)	55.25 ± 7.81	57.00 ± 7.53	53.72 ± 7.85	59.91 ± 7.62	54.16 ± 7.52
Physical domain (4–20)					
Visit 1	14.38 ± 1.78	14.52 ± 1.71	14.26 ± 1.86	15.11 ± 1.64	14.18 ± 1.78
Visit 2	14.97 ± 1.90	15.03 ± 1.89	14.91 ± 1.93	16.00 ± 1.87	14.68 ± 1.82
Visit 3	14.78 ± 1.96	14.98 ± 2.12	14.60 ± 1.82	15.79 ± 1.70	14.54 ± 1.95
Psychological domain (4–20)					
Visit 1	12.64 ± 2.27	13.12 ± 2.04	12.23 ± 2.40	13.46 ± 2.10	12.41 ± 2.29
Visit 2	12.77 ± 2.37	13.25 ± 2.31	12.35 ± 2.37	14.29 ± 2.27	12.34 ± 2.24
Visit 3	12.68 ± 2.48	13.36 ± 1.91	12.09 ± 2.78	14.06 ± 2.56	12.35 ± 2.37
Social domain (4–20)					
Visit 1	14.01 ± 1.85	14.47 ± 1.58	13.63 ± 2.00	14.94 ± 1.48	13.76 ± 1.88
Visit 2	14.27 ± 2.17	14.82 ± 1.98	13.80 ± 2.23	15.69 ± 1.85	13.88 ± 2.09
vVisit 3	13.88 ± 2.16	14.41 ± 1.99	13.42 ± 2.23	15.27 ± 2.00	13.55 ± 2.08
Environment domain (4–20)					
Visit 1	14.02 ± 1.90	14.40 ± 1.91	13.69 ± 1.84	14.83 ± 1.86	13.79 ± 1.86
Visit 2	14.19 ± 2.01	14.43 ± 1.93	13.99 ± 2.08	14.89 ± 2.06	14.00 ± 1.97
Visit 3	13.92 ± 2.13	14.26 ± 2.31	13.62 ± 1.95	14.79 ± 1.97	13.71 ± 2.13
Chi-PCOSQ					
Total scores (6–42)					
Visit 1 (baseline)	24.53 ± 7.61	25.37 ± 7.56	23.82 ± 7.68	27.68 ± 6.50	23.66 ± 7.72
Visit 2 (1st -2nd month of treatment)	25.41 ± 7.33	26.47 ± 7.40	24.51 ± 7.25	28.31 ± 7.41	24.61 ± 7.17
Visit 3(3rd- 4th month of treatment)	25.63 ± 7.21	27.37 ± 7.88	24.12 ± 6.32	28.58 ± 7.60	24.94 ± 7.02
Weight domain (1–7)					
Visit 1	3.25 ± 1.59	3.91 ± 1.58	2.67 ± 1.38	3.69 ± 1.18	3.12 ± 1.68
Visit 2	3.34 ± 1.61	4.17 ± 1.59	2.63 ± 1.25	3.98 ± 1.38	3.16 ± 1.62
Visit 3	3.43 ± 1.49	4.20 ± 1.55	2.76 ± 1.08	3.89 ± 1.25	3.32 ± 1.54
Infertility domain (1–7)					
Visit 1	3.38 ± 1.93	3.27 ± 1.78	3.48 ± 2.07	3.88 ± 2.17	3.25 ± 1.86
Visit 2	3.59 ± 2.01	3.43 ± 1.84	3.73 ± 2.16	3.75 ± 2.36	3.55 ± 1.93
Visit 3	3.76 ± 1.91	3.84 ± 1.85	3.70 ± 2.00	4.00 ± 2.18	3.71 ± 1.87
Menstrual domain (1–7)					
Visit 1	3.67 ± 1.53	3.67 ± 1.48	3.68 ± 1.60	3.55 ± 1.42	3.71 ± 1.57
Visit 2	3.94 ± 1.45	3.98 ± 1.40	3.92 ± 1.50	3.81 ± 1.35	3.98 ± 1.48
Visit 3	3.92 ± 1.31	4.10 ± 1.30	3.76 ± 1.31	4.04 ± 1.38	3.89 ± 1.31
Emotions domain (1–7)					
Visit 1	4.65 ± 1.77	4.72 ± 1.59	4.59 ± 1.97	4.90 ± 1.77	4.58 ± 1.78
Visit 2	4.84 ± 1.65	4.93 ± 1.53	4.76 ± 1.76	5.13 ± 1.69	4.76 ± 1.64
Visit 3	4.79 ± 1.71	5.04 ± 1.78	4.47 ± 1.64	5.05 ± 1.86	4.73 ± 1.69
Body hair domain (1–7)					
Visit 1	5.37 ± 1.60	5.45 ± 1.73	5.30 ± 1.50	6.19 ± 0.99	5.14 ± 1.67
Visit 2	5.38 ± 1.55	5.61 ± 1.56	5.18 ± 1.53	6.20 ± 1.17	5.15 ± 1.57
Visit 3	5.31 ± 1.62	5.67 ± 1.65	5.00 ± 1.54	6.02 ± 1.27	5.14 ± 1.65
Acne & Hair loss domain (1–7)					
Visit 1	4.21 ± 1.63	4.34 ± 1.57	4.10 ± 1.69	5.48 ± 1.16	3.86 ± 1.57
Visit 2	4.32 ± 1.58	4.35 ± 1.66	4.29 ± 1.53	5.44 ± 1.37	4.01 ± 1.50
Visit 3	4.41 ± 1.40	4.50 ± 1.60	4.34 ± 1.22	5.58 ± 1.33	4.14 ± 1.28

PCOS patients had the lowest score on the weight domain of Chi-PCOSQ and the highest score on the body hair domain. This means that weight associated psychological disturbances had the greatest impact on patients’ HRQoL, while patients had less impact of body hair problems on their HRQoL. Overweight patients had significantly lower PCOS-specific HRQoL (relatively poorer PCOS-specific quality of life) as compared to that of normal weight patients. Repeated measures ANOVA indicated that the change in weight domain score along with treatment duration between overweight and normal weight subgroups was significantly different (*p* < 0.0001). Also, hyperandrogenic patients had significantly lower PCOS-specific HRQoL as compared to that of non- hyperandrogenic patients. The change in acne and hair loss domain score along with treatment time between hyperandrogenism and non- hyperandrogenism subgroups was significantly different (*p* < 0.0005), implying that the improved HRQoL, especially on acne and hair loss aspects, was greater in hyperandrogenic patients than that in non-hyperandrogenic patients.

Table 3 shows that the physical domain of WHOQOL-Bref significantly improved with treatment time (*p* = 0.01). Overweight patients had significantly improved physical domain scores during the treatment period, whereas no improvement trend was found for normal weight patients. As for the hyperandrogenism and non-hyperandrogenism subgroups, significantly improved physical domain scores were found only for the former. Table 4 indicates that the infertility and acne and hair loss domains of Chi-PCOSQ significantly improved with treatment time (*p* < 0.05). Overweight patients had significantly improved acne and hair domain scores, and hyperandrogenic patients had significantly improved infertility and acne and hair domain scores.

**Table 3:** Mixed effect model analysis of metformin effect on general HRQoL outcome measured via WHOQOL-Bref

WHOQOL-Bref	Total Coefficient (SE)	Subgroups			
		Normal weight Coefficient (SE)	Overweight Coefficient (SE)	Non- hyperandrogenism Coefficient (SE)	Hyperandrogenism Coefficient (SE)
Total scores					
Treatment time					
Visit 2 (reference = visit 1)	−0.93 (1.04)	0.47 (0.77)	−1.72 (1.44)	1.49 (1.26)	0.79 (0.56)
Visit 3 (reference = visit 1)	0.06 (0.94)	0.07 (0.75)	−0.27 (1.45)	0.52 (1.30)	0.70 (0.56)
Physical domain					
Treatment time	*p = 0.010*		*p = 0.036*		*p = 0.015*
Visit 2 (reference = visit 1)	0.58 (0.20)*	0.50 (0.29)	0.57 (0.28)*	0.62 (0.50)	0.55 (0.22)*
Visit 3 (reference = visit 1)	0.54 (0.20)*	0.50 (0.29)	0.64 (0.27)*	0.29 (0.51)	0.60 (0.22)*
Psychological domain					
Treatment time					
Visit 2 (reference = visit 1)	−0.90 (0.06)	−0.17 (0.34)	−1.49 (0.57)	0.63 (0.58)	−0.16 (0.23)
Visit 3 (reference = visit 1)	−0.06 (0.39)	−0.14 (0.33)	−0.76 (0.57)	0.17 (0.60)	0.002 (0.23)
Social domain					
Treatment time					
Visit 2 (reference = visit 1)	−0.61 (0.39)	0.10 (0.31)	0.18 (0.24)	−0.83 (0.69)	0.05 (0.21)
Visit 3 (reference = visit 1)	−0.31 (0.35)	−0.31 (0.30)	0.14 (0.25)	−0.02 (0.63)	−0.14 (0.21)
Environment domain					
Treatment time					
Visit 2 (reference = visit 1)	0.13 (0.15)	−0.07 (0.23)	0.29 (0.21)	−0.34 (0.27)	0.23 (0.18)
Visit 3 (reference = visit 1)	0.07 (0.16)	−0.06 (0.22)	0.17 (0.22)	−0.16 (0.28)	0.10 (0.18)

Note: Visit 1 is 1st office visit when patients were diagnosed with PCOS (baseline). Once PCOS was diagnosed, patients started metformin treatment. Visit 2 was 2nd office visit (1st-2nd month of treatment). Visit 3 was 3rd office visit (3rd- 4th month of treatment). The analysis was adjusted for the effect of medication adherence via the Morisky 8-item medication adherence scale

*Abbreviations*: *HRQoL* health-related quality of life, *SE* standard error

*p < 0.05

**Table 4:** Mixed effect model analysis of metformin effect on PCOS-specific HRQoL measured via Chi-PCOSQ

Chi-PCOSQ	Total Coefficient (SE)	Subgroups			
		Normal weight Coefficient (SE)	Overweight Coefficient (SE)	Non- hyperandrogenism Coefficient (SE)	Hyperandrogenism Coefficient (SE)
Total scores					
Treatment time					
Visit 2 (reference = visit 1)	0.44 (0.56)	0.75 (0.86)	0.20 (0.75)	−0.58 (0.89)	0.61 (0.66)
Visit 3 (reference = visit 1)	1.25 (0.56)	1.77 (0.84)	0.80 (0.78)	0.11 (0.92)	1.45 (0.66)*
Weight domain					
Treatment time					
Visit 2 (reference = visit 1)	−0.06 (0.14)	0.11 (0.22)	−0.18 (0.19)	−0.04 (0.28)	−0.07 (0.17)
Visit 3 (reference = visit 1)	0.12 (0.15)	0.15 (0.21)	0.10 (0.20)	−0.01 (0.29)	0.15 (0.17)
Infertility domain					
Treatment time	*p = 0.043*				*p = 0.048*
Visit 2 (reference = visit 1)	0.25 (0.18)	0.34 (0.23)	0.15 (0.29)	−0.06 (0.33)	0.32 (0.21)
Visit 3 (reference = visit 1)	0.46 (0.18)*	0.34 (0.24)	0.59 (0.28)*	0.12 (0.34)	0.52 (0.21)*
Menstrual domain					
Treatment time					
Visit 2 (reference = visit 1)	0.10 (0.18)	0.21 (0.29)	0.02 (0.24)	0.26 (0.39)	0.08 (0.20)
Visit 3 (reference = visit 1)	0.19 (0.18)	0.36 (0.27)	0.04 (0.25)	0.58 (0.41)	0.11 (0.20)
Emotions domain					
Treatment time					
Visit 2 (reference = visit 1)	0.07 (0.15)	0.15 (0.21)	0.01 (0.21)	0.82 (0.33)*	0.04 (0.18)
Visit 3 (reference = visit 1)	0.14 (0.15)	0.30 (0.21)	0.003 (0.21)	0.43 (0.29)	0.13 (0.18)
Body hair domain					
Treatment time					
Visit 2 (reference = visit 1)	0.03 (0.14)	0.18 (0.19)	−0.08 (0.20)	−0.34 (0.20)	0.10 (0.17)
Visit 3 (reference = visit 1)	0.02 (0.14)	0.24 (0.18)	−0.16 (0.21)	−0.37 (0.21)	0.10 (0.17)
Acne & Hair loss domain					
Treatment time	*p = 0.008*		*p = 0.043*		*p = 0.007*
Visit 2 (reference = visit 1)	−0.51 (0.28)	−0.10 (0.19)	0.01 (0.20)	−0.46 (0.18)*	−0.55 (0.35)
Visit 3 (reference = visit 1)	0.45 (0.26)	0.08 (0.19)	0.33 (0.21)	−0.28 (0.19)	0.71 (0.32)*

Note: Visit 1 is 1st office visit when patients were diagnosed with PCOS (baseline). Once PCOS was diagnosed, patients started metformin treatment. Visit 2 was 2nd office visit (1st-2nd month of treatment). Visit 3 was 3rd office visit (3rd- 4th month of treatment). The analysis was adjusted for the effect of medication adherence via the Morisky 8-item medication adherence scale

*Abbreviations*: *PCOS* polycystic ovary syndrome, *HRQoL* Health related Quality of life, *SE* standard error

*p < 0.05

## Discussion

There is a lack of research that assesses HRQoL in ethnic Chinese women with PCOS. Also, clinical evidence showing the effect of treatment for PCOS women on HRQoL is scarce. The present study found that psychological disturbances due to PCOS associated problems (i.e., acne, hair loss, infertility) may lead to a reduction in the HRQoL for ethnic Chinese women with PCOS. Overweight and hyperandrogenic patients had significantly lower HRQoL as compared with that of normal weight and non-hyperandrogenic counterparts. Metformin may provide benefits to the HRQoL of PCOS women by ameliorating psychological disturbances due to acne, hair loss and infertility problems, especially for the patients who present overweight and hyperandrogenism.

### HRQoL in PCOS patients treated with metformin

Few studies [7, 21] have assessed treatment effects on HRQoL outcomes for PCOS women. Hahn et al. [21] examined the HRQoL of 64 German women with PCOS with 6-month treatment of metformin. They found positive effects of metformin on HRQoL outcomes, especially on psychosocial, emotional, and psychosexual aspects of well-being. The present study also observed that the emotions domain of HRQoL was generally improved following treatment, although the change was not statistically significant.

Acne and hair loss/androgenetic alopecia could increase women’s self-consciousness, feelings of unattractiveness and emotional distress [10, 11]. Previous studies have shown that metformin alleviates clinical signs of hyperandrogenism (i.e., acne) [32, 33]. However, no studies assessed potential benefits of metformin on HRQoL outcomes associated with the improved clinical signs of hyperandrogenism (i.e., acne). This is in part because acne and hair loss issues are not included in the original PCOSQ [26], which is the most commonly used PCOS-specific HRQoL instrument. The present study used the Chi-PCOSQ, which is a Chinese version of PCOSQ and contains the acne and hair loss domain, and found significantly relieved acne and hair loss associated psychological disturbances (i.e., worried, embarrassed) after metformin treatment. In fact, more than half of study participants having acne or hair loss problem at baseline reported no these problems after 3 – 4 months of treatment, which is even profound in the hyperandrogenism subgroup; 81 % of hyperandrogenism patients without acne or hair loss problems at 3 – 4 months of treatment. Thus, the improved clinical signs of hyperandrogenism as a result of metformin may lead to satisfactory HRQoL of PCOS patients.

Previous research has supported that insulin sensitizers (e.g., metformin) provide fertility benefits to PCOS patients (i.e., improve pregnancy rate [16]), especially those with hyperinsulinemia or insulin resistance, which could be responsible for the abnormal ovarian response [34]. Guyatt et al.’s study observed significant improvements in the infertility aspect of the PCOS-specific HRQoL (measured via PCOSQ) after 44 weeks of troglitazone treatment [7]. Troglitazone, an insulin sensitizer like metformin, was previously used in PCOS. The present study also observed improved infertility aspect of PCOS-specific HRQoL, especially in hyperandrogenic patients. Thus, the amelioration of ovulation problems achieved by insulin sensitizers (i.e., troglitazone, metformin) may alleviate emotional distress due to infertility and thus contribute to the improvement of the HRQoL of PCOS patients. The infertility domain in Chi-PCOSQ consists of three items: “concerned about infertility problems”, “afraid of not being able to have children”, and “sad because of infertility problems”. These items are associated with patient’s psychological concerns about fertility. Although we did not find a significantly increase pregnancy rate in our reproductive-aged participants after treatment, patient’s perceived benefits from metformin and reduced perceived susceptibility to infertility after treatment [35] may lead to the improved fertility aspect of HRQoL that we observed.

Metformin is recommended as one of treatment options in PCOS women, especially for those who present obesity, hyperandrogenism, insulin resistance or hyperinsulinemia [18]. Consistently, our results showed significant effects of metformin on HRQoL of PCOS patients, especially in overweight and hyperandrogenic patients. Also, insulin resistance is one of important characteristics for metformin efficacy [33]. Our previous study showed that with metformin treatment, overweight PCOS women (BMI ≥ 25 kg/m^2^) had a significant reduction in body weight as compared to those with normal weight and patients with insulin resistance had a significantly improved 2-h insulin level as compared to those without insulin resistence [19]. In the present study, we found that the prevalence of insulin resistance in the overweight group was higher than that in the normal weight group (85 versus 58 %). This may be another reason why positive effect of metformin on HRQoL outcomes was observed in overweight patients, but not in normal weight patients. Moreover, it has been argued that the combination of menstrual problems, hyperandrogenism and anovulation can be positively affected by metformin treatment [18]. This may explain our findings showing that metformin provided significant benefits in HRQoL outcomes for PCOS women with hyperandrogenism, especially in terms of mitigating the burden of acne and hair loss, and infertility associated psychological distress on the HRQoL of patients.

### Importance of study findings to clinicians and patient care

Considerable burden of psychological disturbances due to acne, hair loss and infertility problems on the HRQoL of ethnic Chinese women with PCOS requires healthcare providers’ attention. Regularly assessing the HRQoL of PCOS patients through generic and/or disease-specific HRQoL instruments would help clinicians detect any changes in patients’ HRQoL due to clinical signs of PCOS or treatment interventions. As a supplement to lifestyle changes (e.g., exercise and weight control), the amelioration of clinical signs of PCOS achieved by metformin may lead to improvement in HRQoL. Also, PCOS patients who present overweight or hyperandrogenism may receive the most benefit from metformin treatment on their HRQoL.

### Potential limitations

Several limitations of this study need to be addressed. First, all participants were from one medical center in southern Taiwan. Our findings may be applicable to only a subset of Chinese women. Ethnically Chinese people are distributed over a large geographic area, and are likely to have differences in dietary habits, physical activity, and even treatment approaches. Second, all participants knew that they were receiving metformin and thus, the improved HRQoL which we observed may be in part because of their motivation to improve. Third, because this was an observational study and all participants received metformin treatment after PCOS diagnosis, this study did not compare other treatments for PCOS (e.g., oral contraceptives, as active control) or have a placebo control group. However, other types of treatment (e.g., clomiphene) for PCOS may also provide benefits (e.g., reproductive) for the HRQoL of patients. Fourth, because this was not a randomized design study, selection bias could not be avoided. Moreover, our results were based on patients’ reporting of HRQoL outcomes, and thus, self-reporting bias could not be avoided. Lastly, the present study did not include objective measures (e.g., pregnancy rate) as indicators for metformin treatment. So, improvement in subjective outcomes (e,g, HRQoL) may not be explained as the result of changes in objective measures of clinical outcomes. However, we did observed clinical improvement in patient’s body weight and acne and hair loss problems.

## Conclusions

This is the first study to apply Chi-PCOSQ to assess the HRQoL of ethnic Chinese with PCOS and to evaluate the HRQoL outcomes of PCOS patients after metformin treatment. The results provide important clinical implications for the care of PCOS patients and suggest that developing interventions for improving the HRQoL of PCOS patients is needed. Future studies from other countries/ethnicity are warranted to evaluate the influence of treatment on the HRQoL outcomes of PCOS women.

## Abbreviations

Chi-PCOSQ: Chinese version of health-related quality-of-life questionnaire for women with polycystic ovary syndrome
HRQoL: Health-related quality of life ovary syndrome
PCOS: Polycystic ovary
PCOSQ: Health-related quality-of-life questionnaire for women with polycystic
WHOQOL: World Health Organization Quality of Life
WHOQOL-Bref: An abbreviated version of World Health Organization Quality of Life
