# Erratum to: Metformin improved health-related quality of life in ethnic Chinese women with polycystic ovary syndrome

**DOI:** 10.1186/s12955-016-0528-1

**Published:** 2016-09-22

**Authors:** Huang-Tz Ou, Pei-Chi Chen, Meng-Hsing Wu, Chung-Ying Lin

**Affiliations:** 1 Institute of Clinical Pharmacy and Pharmaceutical Sciences, College of Medicine, National Cheng Kung University, 1 University Road, Tainan, 7010 Taiwan; 2 Department of Obstetrics and Gynecology, College of Medicine, National Cheng Kung University and Hospital, Tainan, Taiwan; 3 Department of Rehabilitation Sciences, Faculty of Health and Social Sciences, The Hong Kong Polytechnic University, Hung Hom, Hong Kong

## Erratum

The original version of this article [1] contained a typing error in the first author’s name, showing it mistakenly as “Huang-TzOu”.

The author’s name has now been updated in the original article to the correct spelling of “Huang-Tz Ou”.
