# Correction to: Metformin improved health-related quality of life in ethnic Chinese women with polycystic ovary syndrome

**DOI:** 10.1186/s12955-018-1026-4

**Published:** 2018-11-19

**Authors:** Huang-Tz Ou, Pei-Chi Chen, Meng-Hsing Wu, Chung-Ying Lin

**Affiliations:** 1 Institute of Clinical Pharmacy and Pharmaceutical Sciences, College of Medicine, National Cheng Kung University, 1 University Road, Tainan, 7010 Taiwan; 2 Department of Obstetrics and Gynecology, College of Medicine, National Cheng Kung University and Hospital, Tainan, Taiwan; 3 Department of Rehabilitation Sciences, Faculty of Health and Social Sciences, The Hong Kong Polytechnic University, Hung Hom, Hong Kong

## Correction

The original article [1] contains a number of statements that the authors would like to be disregarded, noted ahead:

1. In the Methods section, along with the references mentioned in the statement:
  ‘In order to account for the effect of medication behavior, the Morisky 8-item medication adherence scale (MMAS-8) [30] was applied to measure medication adherence. The MMAS-8 is one of the most commonly used self-report adherence questionnaires. The Chinese version of MMAS-8 has been validated among a convenience sample of 176 patients in China [31]. The scale showed acceptable internal consistency (Cronbach’s α = 0.77) and test-retest reliability (r = 0.88), and good construct validity.’
2. In the respective captions of Tables 3 and 4:
  ‘The analysis was adjusted for the effect of medication adherence via the Morisky 8-item medication adherence scale’.

**Table 3:** Mixed effect model analysis of metformin effect on general HRQoL outcome measured via WHOQOL-Bref

WHOQOL-Bref	Total Coefficient (SE)	Subgroups			
		Normal weight Coefficient (SE)	Overweight Coefficient (SE)	Non- hyperandrogenism Coefficient (SE)	Hyperandrogenism Coefficient (SE)
Total scores					
Treatment time					
Visit 2 (reference = visit 1)	−0.93 (1.04)	0.47 (0.77)	−1.72 (1.44)	1.49 (1.26)	0.79 (0.56)
Visit 3 (reference = visit 1)	0.06 (0.94)	0.07 (0.75)	−0.27 (1.45)	0.52 (1.30)	0.70 (0.56)
Physical domain					
Treatment time	*p = 0.010*		*p = 0.036*		*p = 0.015*
Visit 2 (reference = visit 1)	0.58 (0.20)*	0.50 (0.29)	0.57 (0.28)*	0.62 (0.50)	0.55 (0.22)*
Visit 3 (reference = visit 1)	0.54 (0.20)*	0.50 (0.29)	0.64 (0.27)*	0.29 (0.51)	0.60 (0.22)*
Psychological domain					
Treatment time					
Visit 2 (reference = visit 1)	−0.90 (0.06)	−0.17 (0.34)	−1.49 (0.57)	0.63 (0.58)	−0.16 (0.23)
Visit 3 (reference = visit 1)	−0.06 (0.39)	−0.14 (0.33)	−0.76 (0.57)	0.17 (0.60)	0.002 (0.23)
Social domain					
Treatment time					
Visit 2 (reference = visit 1)	−0.61 (0.39)	0.10 (0.31)	0.18 (0.24)	−0.83 (0.69)	0.05 (0.21)
Visit 3 (reference = visit 1)	−0.31 (0.35)	−0.31 (0.30)	0.14 (0.25)	−0.02 (0.63)	−0.14 (0.21)
Environment domain					
Treatment time					
Visit 2 (reference = visit 1)	0.13 (0.15)	−0.07 (0.23)	0.29 (0.21)	−0.34 (0.27)	0.23 (0.18)
Visit 3 (reference = visit 1)	0.07 (0.16)	−0.06 (0.22)	0.17 (0.22)	−0.16 (0.28)	0.10 (0.18)

Note: Visit 1 is 1st office visit when patients were diagnosed with PCOS (baseline). Once PCOS was diagnosed, patients started metformin treatment. Visit 2 was 2nd office visit (1st-2nd month of treatment). Visit 3 was 3rd office visit (3rd- 4th month of treatment). The analysis was adjusted for the effect of medication adherence via the Morisky 8-item medication adherence scale

*Abbreviations*: *HRQoL* health-related quality of life, *SE* standard error

*p < 0.05

**Table 4:** Mixed effect model analysis of metformin effect on PCOS-specific HRQoL measured via Chi-PCOSQ

Chi-PCOSQ	Total Coefficient (SE)	Subgroups			
		Normal weight Coefficient (SE)	Overweight Coefficient (SE)	Non- hyperandrogenism Coefficient (SE)	Hyperandrogenism Coefficient (SE)
Total scores					
Treatment time					
Visit 2 (reference = visit 1)	0.44 (0.56)	0.75 (0.86)	0.20 (0.75)	−0.58 (0.89)	0.61 (0.66)
Visit 3 (reference = visit 1)	1.25 (0.56)	1.77 (0.84)	0.80 (0.78)	0.11 (0.92)	1.45 (0.66)*
Weight domain					
Treatment time					
Visit 2 (reference = visit 1)	−0.06 (0.14)	0.11 (0.22)	−0.18 (0.19)	−0.04 (0.28)	−0.07 (0.17)
Visit 3 (reference = visit 1)	0.12 (0.15)	0.15 (0.21)	0.10 (0.20)	−0.01 (0.29)	0.15 (0.17)
Infertility domain					
Treatment time	*p = 0.043*				*p = 0.048*
Visit 2 (reference = visit 1)	0.25 (0.18)	0.34 (0.23)	0.15 (0.29)	−0.06 (0.33)	0.32 (0.21)
Visit 3 (reference = visit 1)	0.46 (0.18)*	0.34 (0.24)	0.59 (0.28)*	0.12 (0.34)	0.52 (0.21)*
Menstrual domain					
Treatment time					
Visit 2 (reference = visit 1)	0.10 (0.18)	0.21 (0.29)	0.02 (0.24)	0.26 (0.39)	0.08 (0.20)
Visit 3 (reference = visit 1)	0.19 (0.18)	0.36 (0.27)	0.04 (0.25)	0.58 (0.41)	0.11 (0.20)
Emotions domain					
Treatment time					
Visit 2 (reference = visit 1)	0.07 (0.15)	0.15 (0.21)	0.01 (0.21)	0.82 (0.33)*	0.04 (0.18)
Visit 3 (reference = visit 1)	0.14 (0.15)	0.30 (0.21)	0.003 (0.21)	0.43 (0.29)	0.13 (0.18)
Body hair domain					
Treatment time					
Visit 2 (reference = visit 1)	0.03 (0.14)	0.18 (0.19)	−0.08 (0.20)	−0.34 (0.20)	0.10 (0.17)
Visit 3 (reference = visit 1)	0.02 (0.14)	0.24 (0.18)	−0.16 (0.21)	−0.37 (0.21)	0.10 (0.17)
Acne & Hair loss domain					
Treatment time	*p = 0.008*		*p = 0.043*		*p = 0.007*
Visit 2 (reference = visit 1)	−0.51 (0.28)	−0.10 (0.19)	0.01 (0.20)	−0.46 (0.18)*	−0.55 (0.35)
Visit 3 (reference = visit 1)	0.45 (0.26)	0.08 (0.19)	0.33 (0.21)	−0.28 (0.19)	0.71 (0.32)*

Note: Visit 1 is 1st office visit when patients were diagnosed with PCOS (baseline). Once PCOS was diagnosed, patients started metformin treatment. Visit 2 was 2nd office visit (1st-2nd month of treatment). Visit 3 was 3rd office visit (3rd- 4th month of treatment). The analysis was adjusted for the effect of medication adherence via the Morisky 8-item medication adherence scale

*Abbreviations*: *PCOS* polycystic ovary syndrome, *HRQoL* Health related Quality of life, *SE* standard error

*p < 0.05

These statements as well as the references contained within them are to be disregarded as it was discovered that the patients included in the analyses did not receive the MMAS and thus, the analyses did not adjust for the MMAS scores (as was erroneously asserted in the original text).
